# A Journey into Animal Models of Human Osteomyelitis: A Review

**DOI:** 10.3390/microorganisms10061135

**Published:** 2022-05-31

**Authors:** Gabriele Meroni, Alexios Tsikopoulos, Konstantinos Tsikopoulos, Francesca Allemanno, Piera Anna Martino, Joel Fernando Soares Filipe

**Affiliations:** 1 One Health Unit, Department of Biomedical, Surgical, and Dental Sciences, University of Milan, Via Pascal 36, 20133 Milan, Italy; allemannofrancesca@gmail.com (F.A.); piera.martino@unimi.it (P.A.M.); 2 Department of Pharmacology, School of Medicine, Faculty of Health Sciences, Aristotle University of Thessaloniki, 541 24 Thessaloniki, Greece; alextsikop@yahoo.gr; 3 Orthopaedic Department, 404 Army Hospital, 412 22 Larissa, Greece; kostastsikop@gmail.com; 4 Department of Veterinary Medicine and Animal Sciences, University of Milan, Via dell’Università 6, 26900 Lodi, Italy; joel.soares@unimi.it

**Keywords:** osteomyelitis, animal models, orthopedics, microbiology, immunology

## Abstract

Osteomyelitis is an infection of the bone characterized by progressive inflammatory destruction and apposition of new bone that can spread via the hematogenous route (hematogenous osteomyelitis (HO)), contiguous spread (contiguous osteomyelitis (CO)), and direct inoculation (osteomyelitis associated with peripheral vascular insufficiency (PVI)). Given the significant financial burden posed by osteomyelitis patient management, the development of new preventive and treatment methods is warranted. To achieve this objective, implementing animal models (AMs) of infection such as rats, mice, rabbits, avians, dogs, sheep, goats, and pigs might be of the essence. This review provides a literature analysis of the AMs developed and used to study osteomyelitis. Historical relevance and clinical applicability were taken into account to choose the best AMs, and some study methods are briefly described. Furthermore, the most significant strengths and limitations of each species as AM are discussed, as no single model incorporates all features of osteomyelitis. HO’s clinical manifestation results in extreme variability between patients due to multiple variables (e.g., age, sex, route of infection, anatomical location, and concomitant diseases) that could alter clinical studies. However, these variables can be controlled and tested through different animal models.

## 1. Background

The use of animal models in medicine is an ancient story, with the first written records dated to 2000 BC as stated in the book “Laboratory Animal Medicine” (2nd edition, chapter 30, page 1206) “*The earliest written records of animal experimentation date to 2000 BC when Babylonians and Assyrians documented surgery and medications for humans and animals*” [1]. From this written milestone, animals as models to mimic human diseases were implemented mainly to resemble, more precisely, human pathology. The use of models is nowadays strictly regulated to avoid unnecessary suffering of the animals (ethical concerns), ensuring respect for animal welfare and the development of alternative methods in compliance with current European legislation (DIRECTIVE 2010/63/EU on the protection of animals used for scientific purposes) [2]. The need for animal models in orthopedics allowed the development of new therapeutic approaches and the refinement of traditional surgical methods to a new concept of personalized medicine [3,4,5]. This review describes the history of using animal models in orthopedics to mimic human osteomyelitis with a critical description of the disease’s microbiological basis and immunological characteristics. PubMed was used as a reference point to search for articles over more than 120 years (1900 to December 2021) on large and small animal models.

## 2. Materials and Methods

A literature analysis was accomplished in the PubMed database by considering published articles in English from 1900 until December 2020. The search query was executed by including the following keywords: “animal models”, “mouse”, “rat”, “rabbit(s)”, “poultry”, “dog(s)”, “pig(s)”, “osteomyelitis”, “hematogenous osteomyelitis”, “contiguous osteomyelitis”, “orthopedic infection(s)”, “bone infection(s)”, “biofilm-related infection”. Eligibility criteria were used to identify non-pertinent studies and focus only on studies exploring animal models for human osteomyelitis. On the other hand, unpublished literature, in vitro studies, non-orthopedic-oriented articles, book chapters, and papers published in another language were discarded. The year of publication was not considered a disregarding factor since this review focused on the history of using animals as experimental models to study osteomyelitis.

## 3. Bacterial Biofilm

The National Institutes of Health (NIH) advertised that among all microbial and chronic infections, 65% and 80%, respectively, are related to biofilm formation, underlining their extreme difficulty in treatment [6,7,8]. Furthermore, Jamal estimated the prevalence of device-related infections for several devices, being 2% for breast implants; 2% for joint prostheses; 4% for mechanical heart valves; 10% for ventricular shunts; 4% for pacemakers and defibrillators, and about 40% for ventricular-assisted devices [7].

Biofilm (both monomicrobial and polymicrobial) is an organized ensemble of microorganisms living within a self-produced extracellular polymeric matrix and irreversibly attached to an abiotic or biotic surface [7,9,10]. The formation and accumulation of extracellular polymeric substances (EPS) start at the beginning of the biofilm formation process. Then, planktonic bacteria adhere to a surface, starting a complete transcriptomic and metabolic shift to a sessile form [11,12,13]. The thickness of the EPS matrix is usually 20–40 nm, and the biofilm size does not exceed 5–88 µm [14]. The biofilm matrix is mainly composed of different substances: protein (>2%), polysaccharides (1–2%); extracellular DNA (<1%), RNA (<1%); ions (bound and free), and ±97% of water [6,7,13,15,16]. Due to its extreme and complex architecture, an intricate water channel system ensures the flow of essential nutrients inside a biofilm [17,18]. At least two properties are required for bacteria to create a biofilm: a communication system (called “*quorum sensing*”) [19,20,21,22,23] and a complex set of genes different from those commonly expressed during planktonic life [19,24,25,26,27]. Biofilm formation is complex but occurs in a few common steps: (i) attachment to the surface, (ii) micro-colony formation, (iii) maturation and formation of the architecture of the biofilm, and (iv) dispersion [8,28]. In the initial phase, microbial cells adhere to the biotic/abiotic surface through mechanical forces, like pili and/or flagella, physical interactions like van der Waal’s forces, and electrostatic exchanges [13]. Fimbriae, pili, and flagella are highly complex structures that help bacteria give strength to the biofilm acting as a point of connection between bacteria and the surface [29,30]. The adhesion step is followed by a cohesion phase in which bacteria cross-talk via *quorum sensing* and link one another [7]. The specific characteristics of a surface (e.g., hydrophobicity, roughness, surface charge) and those of the surrounding environment (liquid flow speed, pressure, osmolarity, pH) are crucial in reinforcing microbes’ attachment and reducing the repulsive forces between bacteria and surface [31,32]. For these reasons, non-polar surfaces (e.g., plastic) are more likely colonized by bacteria rather than polar surfaces (e.g., metal and glass) [33,34,35]. After the attachment to the surface, the multiplication and division of microbial cells start. Micro-colonies can occur via the production and secretion of chemical signals that are different between Gram-positive (small peptides, also known as auto-inducing peptides or AIPs) and Gram-negative (lipids). These chemical modulators will coordinate the exchange of nutrients and small fragments of DNA through the *quorum-sensing* system. During biofilm maturation, bacteria show the most complex communication system, based on auto-inducers molecules’ production, strictly dependent on cellular density. At this stage, bacteria can control the proliferation and delivery of nutrients. As the biofilm matrix envelops bacteria and protects them from external stimuli, there is a progressive decrease in nutrient and oxygen availability [18]. To overcome this problem, bacteria act at two levels, rearrangement of cells in a metabolic-dependent way (e.g., anaerobes in the inner core and aerobes at the distal surface) and transcriptomic shift (e.g., quiescent cells at the interface with the surface and active bacteria in the outer part of architecture) [28,36]. The last phase of biofilm formation is characterized by the rapid enzymatic remodeling of the biofilm’s external layer, leading to the dispersion of bacterial cells, which rapidly convert from sessile into motile form. The production of specific enzymes is dependent on the specific EPS produced (e.g., *Escherichia coli* produces N-acetyl-heparosan lyase, *Pseudomonas aeruginosa*, and *Pseudomonas fluorescents* produce alginate lyase, and *Streptococcus equi* produces hyaluronidase) [7].

### 3.1. Device-Related and Non-Device-Related Biofilm Infections

The ability of bacteria to colonize almost all surfaces gives them the possibility to cause severe infections resulting in therapy difficulties and economic loss. Device-related infections (e.g., osteomyelitis) primarily originate from bacteria on patients’ skin during implant insertion and then reach the device surface from the incision site or by hematogenous seeding, starting the biofilm formation [37]. Contact lenses, central venous catheters, mechanical heart valves, peritoneal dialysis catheters, prosthetic joints, pacemakers, urine catheters, and vocal prostheses are suitable bacteria targets. [7,20,38]. Non-device-related bacterial biofilm infections include dental plaque, cystic fibrosis, osteomyelitis, periodontitis, and chronic inflammatory diseases [8]. The mouth can be colonized by up to 500 species of bacteria [39]. The following bacterial species were seen in osteomyelitis of the jawbones *Streptococcus* spp., *Eikenella* spp., *Staphylococcus* spp., *Actinomyces* spp., *Klebsiella* spp., *Lactobacillus* spp., *Haemophilus* spp. [40], and anaerobic bacteria, such as *Bacteroides* spp., *Peptostreptococcus* spp., and *Fusobacterium* spp. [39]. The colonization of dental surface follows specific steps; Gram-positive cocci are the primary colonizers (*Streptococcus mutans*, *Streptococcus mitis*, *Streptococcus sanguinis*, *Streptococcus oralis*), followed by Gram-positive rods, Actinobacteria (*A. israelii* and *A. viscosus*), and several Gram-negative cocci [41]. Gram-positive facultative anaerobic bacteria are the most abundant biofilm producers in healthy adults, while, during chronic gingivitis, the number of Gram-negative anaerobic bacteria increases about ten times that of commensal species found in healthy individuals [8]. L-form bacterial pathogens and biofilms can cause chronic inflammatory disorders [42]. The mode of action to induce chronic disorders resides in the production of molecules that attach and inactivate the vitamin D receptors; these proteins modulate the activity of the innate immune cells [8].

### 3.2. Microbiology of Bacterial Osteomyelitis

From a microbiological point of view, most HO is generally monomicrobial [8,43,44], but few clinical cases are caused by more than one microorganism [45]. The most isolated pathogen (80% of culture-positive cases) from clinically relevant HO is *S. aureus*, followed by group A *Streptococci* (GAS) [8,43,44]. However, the bacterial etiology of osteomyelitis varies with the individual age [44]. The incidence is approximately 8 cases per 100,000 children (<5 years) per year [46]. In infants, *S. aureus*, *S. agalactiae*, and *E. coli* represent the most frequently isolated microorganisms, while in children, *S. aureus*, *S. pyogenes*, and *Haemophilus influenzae* [43]. In a retrospective study on 167 children in Malawi, Beckles (2010) reported the prevalence of causative agents found in microbiological examinations as follows “*61% episodes were caused by *S. aureus*, 4% by E. coli, 2% by Streptococcus, 2% by Pseudomonas, 1% by Bacillus subtilis, 1% by Proteus and in 29% no microorganism could be detected*” [47,48]. In addition to *S. aureus*, *Salmonella* spp. has to be noted as a potential causative agent of the disease; however, with a low prevalence (0.45% of osteomyelitis infections) [49,50]. Beyond the “common” bacteria, the identification of *Kingella kingae* as a new potential aetiological agent is rapidly increasing, especially in young children (excluding the neonatal period), with a prevalence of up to 82% [51,52]. Historically, one of the most persistent pathogens in children is *Haemophilus influenzae* type B. Retrospective research conducted in Canada prior to the implementation of immunization programs, the incidence of this pathogen-related osteomyelitis was estimated to be around 5%.[52]. However, after starting vaccination programs in the 1990s, its prevalence drastically decreased [46]. Rare causes of osteomyelitis in pediatric patients are *M. tuberculosis*, *Bartonella henselae*, and fungi (e.g., *Histoplasma* spp. and *Cryptococcus* spp.) [53,54].

*S. aureus* is the most common causative organism of this disease, responsible for 66% to 70% of cases [55]. Among different strains, community-acquired methicillin-resistant *S. aureus* (CA-MSRA) is the new rapidly spreading bacterium with isolation prevalence strictly dependent on the geographical areas. CA-MRSA was the causative agent of osteomyelitis in 30–40% of pediatric infections [44,55].

In adults, HO is rare and most frequently involves the vertebral bodies [56]. Beyond the well-recognized role of *S. aureus*, some other pathogens are thought to have the same ability, including *Enterococcus* spp., *Streptococcus* spp., *P. aeruginosa*, *Enterobacter* spp., *Mycobacterium* spp., as well as anaerobic bacteria and fungi (e.g., *Candida* spp.) [8,43]. In a study by Aytaç (2014), among 67 microbiological examinations of patients with osteomyelitis, 33% were caused by coagulase-negative staphylococci (CNS), 30% by *S. aureus*, 21% by Gram-negative bacilli, and 19% by *Enterococcus* spp. [57].

In the elderly, which are commonly subject to bacteremia (frequently by Gram-negative bacteria), vertebral osteomyelitis is more frequent than the hematogenous type and is caused by Gram-negative bacilli [56,58].

It is estimated that up to 15% or more of patients with diabetes will develop foot problems during their lifetime [56]. Polymicrobial communities, also embedded in biofilm, are found in diabetic foot osteomyelitis patients, including *S. aureus*, CNS, *Streptococcus* spp., *Enterococcus* spp., Gram-negative bacilli, and anaerobes [43,56].

Despite the monomicrobial community that characterizes HO, multiple microorganisms can usually be recognized in contiguous focus osteomyelitis; among this complex and dynamic community, *S. aureus* and CNS still have a pivotal role in the bacterial ecosystem with a prevalence of 31–47% among bacterial isolates, followed by *Streptococcus* (27–61%), and Gram-negative enteric bacteria (20–50%) [58].

## 4. Immune Response

The innate immune response is one of the first weapons against pathogens, and it acts through the identification of a broad range of microbial components (e.g., nucleic acids, cell wall constituents) that can be recognized by specific receptors on the cells surface (e.g., polymorphonuclear neutrophils (PMNs), dendritic cells (DCs), macrophages) [59,60].

The first line of defense at the site of infection are PMNs that, moving from the bloodstream, can detect and fight both planktonic bacteria and bacterial biofilms and recruit monocytes/macrophages and modulate their activity [61,62,63]. PMNs chemotaxis is activated by different chemo-attractant molecules (cytokines and chemokines), and their primary function is to phagocytize and generate reactive oxygen species (ROS) against opsonized bacteria. However, when talking about biofilm, PMNs efficiency is highly dependent on its maturation state [64,65]. For example, Stroh et al. demonstrated that biofilm opsonization with neither immunoglobulin G nor complement did improve the degree of cellular adherence of PMNs to the biofilm surface, facilitated degranulation, or inducted phagocytosis in vitro. On the other hand, the generation of ROS was critically dependent on the biofilm opsonization with immunoglobulin G [66].

Monocytes and macrophages are also known to phagocyte planktonic bacteria. However, their impact on bacterial biofilms is still unclear. There is evidence that *S. aureus* biofilms can change macrophage phagocytosis efficiency and bactericidal action in different ways, helping, for example, to shield *S. aureus* cells from Toll-Like Receptors (TLRs) recognition and consequent macrophages activation. In addition, thanks to their great size, bacteria are difficult to gulp when clustered in biofilms, even for professional phagocytic cells such as macrophages [67].

Specific immune responses to biofilm bacteria have yet to be discovered when looking for the key distinctions in immune responses to planktonic or biofilm bacteria; the only obvious difference is the difficulty of removing the bacteria when biofilm is present, causing the infection to become chronic [68]. However, as far as bacterial biofilms are concerned, the presence of lactoferrin has been revealed to be of particular importance, as it can suppress the formation of biofilm for some staphylococcal species [69,70].

Whenever there is a bacterial infection with the production of a biofilm, it also leads to the activation of T-cells and monocytes, which is traduced in a local increase of proinflammatory cytokines (e.g., Tumour Necrosis Factor-α (TNF-α), interleukin (IL)-1, interleukin (IL)-6) [64,66,71,72,73]. Unfortunately, sometimes, and for reasons still not completely understood, this continuous release of inflammatory mediators does not help control the infection and spreads into osteolytic and tissue-damaging processes [71].

The presence of bacteria activates immune cells and directly impacts bone tissue, in which cellular components increase the expression of Toll-like receptor 2 (TLR2). For example, when in contact with *S. aureus*, not only the TLR2 expression but also the activity level of Jun N-terminal kinases (JNK) seem to directly impact osteoblast apoptosis and osteogenic differentiation after the bacterial invasion [74,75]. Furthermore, it has recently been shown that bacterial endotoxins, especially lipopolysaccharide (LPS), can also promote cell apoptosis and inhibit the differentiation of osteoblasts by JNK pathway activation [76]. These findings were confirmed in an in vitro study, suggesting that methicillin-resistant *S. aureus* biofilms liberate soluble molecules capable of decreasing osteoblasts viability and osteogenic potential, indirectly promoting osteoclast activity [77].

In terms of cytokines, those from the TNF superfamily promote apoptotic cell signaling in a variety of cell types (including osteoblasts) via a direct interaction between the soluble TNF-related apoptosis-inducing ligand (TRAIL) and specific cell receptors, such as osteoprotegerin (OPG), which acts as a decoy soluble receptor for TRAIL and the receptor activator of nuclear factor-kappa-β ligand (RANKL), promoting osteoclastogenesis [78,79,80].

As part of the immune system, osteoblasts may create antimicrobial peptides in response to bacterial invasion, with human defensins being one of these antimicrobial peptides that can be extremely effective in combatting a wide range of pathogens. [81,82]. The expression of β-defensins in bone tissues has already been demonstrated in both healthy and infected bone, concluding that antimicrobial peptides might also play a role in osteomyelitis [83,84]. Furthermore, a decrease in antimicrobial peptide expression was found after administering immunosuppressive drugs, which was expected to modulate the susceptibility to osteomyelitis [85].

Osteomyelitis is usually associated with high levels of pro-inflammatory cytokines (e.g., TNFα, IL-1β, IL-1α, and IL-6), that have been shown to promote osteoclast formation in vitro, both directly by stimulating bone-resorbing osteoclasts and indirectly by promoting osteoblast production of RANKL to drive osteoclastogenesis [86,87,88,89,90].

On the other hand, MyD88 is an essential component of the innate immune system by communicating with IL-1R and TLRs. In part, thanks to the capability of IL-1 to intermediate the neutrophil enrollment and to endorse the formation of a fibrous capsule (abscess) capable of containing the bacteria (e.g., *S. aureus*) [91,92]. Moreover, especially in the bone marrow, IL-1 is also responsible for promoting granulopoiesis [93,94] and has an already known role in anti-staphylococcal immunity and compelling evidence that IL-1 signaling is capable of affecting bone cells in vitro. Putman et al., 2019, demonstrated that MyD88 and IL-1R signaling are necessary for efficient antibacterial immune responses during osteomyelitis but, on the other hand, may also promote osteoclastogenesis and host-mediated bone loss during osteomyelitis [95].

## 5. Elements of Bone Anatomy

Bone is a composite material and specialized connective tissue which consists of organic and inorganic components [96]. From a functional point of view, bone is the primary reservoir of calcium and phosphate in the body. On top of that, bone contributes to hematopoiesis and plays a protective and mechanical role. It has cellular components whose active constituents are devoted to the production and rearrangement of the osteoid matrix. There are seven cell types in the bone: (1) progenitor bone cells, (2) osteoblasts, (3) osteocytes, (4) osteoclasts, (5) hematopoietic cells, (6) vasculature, and (7) endosteal cells. Concerning bone formation steps, vascular buds first invade the mesenchymal model. Osteoprogenitor cells then migrate through vascular buds and differentiate into osteoblasts giving rise to primary ossification centers and, as a result, cartilage model forms. Subsequently, growth happens appositionally and interstitially. Of note, bone marrow forms by resorption of a central portion of the cartilage, and secondary ossification centers develop at bone ends, thus leading to growth plates. Osteocytes derive from osteoblasts that remain imprisoned in the calcified matrix after synthesis. [97,98]. The matrix has an organic component consisting of collagen fibers, chondroitin sulfate, amorphous substance (e.g., proteoglycans and glycoproteins), and an inorganic component consisting mainly of calcium phosphate and carbonate.

The mature bone tissue has a lamellar structure. The *lamellae* are aggregated into parallel layers, and each *lamella* is formed by cells and the extracellular matrix. The osteon is the morphofunctional unit of the bone. It is made of concentric *lamellae* around the Haversian canal containing arteries, veins, and nerves. Instead, the canaliculi and the canals of Volkmann (arranged transversely or obliquely) allow communication between osteocytes and osteons, respectively.

### 5.1. Histopathological Generalities on Osteomyelitis

When bone tissue becomes infected, bacteria induce an acute inflammatory reaction, with infiltration of neutrophils and systemic symptoms. Bacteria and inflammation affect the periosteum, the blood supply decreases, and the bacteria spread inside the bone, causing necrosis [56,99].

In children, the periosteum is not entirely attached to the cortex, which causes a subperiosteal abscess to form on the bone surface. In addition, the lifting of the periosteum further compromises blood flow to the bone, causing segmental bone necrosis, known as “*sequestrum*” [100].

In chronic osteomyelitis, numerous inflammatory cells and the released cytokines stimulate bone resorption by osteoclasts, fibrous tissue growth, and deposition of new bone tissue in the periphery. When new bone tissue deposits, a sleeve (called “*involucrum*”) around the devitalized bone is formed, and the rupture of the subperiosteal abscess can lead to a soft tissue abscess and possibly form a “draining sinus” [100,101]. Clinically, the gold standard method for diagnosing osteomyelitis remains the bone biopsy coupled with histopathologic examination and tissue culture [58].

### 5.2. Periprosthetic Joint Infection (PJI)

Most PJIs are caused by intra-operative contamination, which can result in early or delayed infection, whereas hematogenous seeding is less common, with late infections occurring years later. Both early postoperative and hematogenous infections have an acute onset, despite their different pathogenesis. On the other hand, chronic late infections can be caused by less virulent microorganisms, and while they are thought to be caused by intraoperative contamination, symptoms appear slowly. Although there is no consistent definition in the literature, most authors agree on classification: early = 3–6 weeks, chronic = 6 weeks, and beyond. This 3–6 week cut-off aims to discriminate against patients requiring DAIR vs. 2-stage revision arthroplasty [101,102]. Early infections can result from perioperative bacterial inoculation, while delayed infections may result from less virulent bacteria inoculation or hematogenous sources. Late infections are commonly caused by an infection carried by the blood to the prosthesis site [101,102,103]. New major criteria for diagnosing PJI were drawn out by Parvizi (2018) as two positive cultures or the presence of a sinus tract. Giving score values for specific clinical parameters (e.g., elevated serum CRP (>1 mg/dL), D-dimer (>860 ng/mL), erythrocyte sedimentation rate (>30 mm/h), elevated synovial fluid white blood cell count (>3000 cells/μL), α-defensin (signal-to-cutoff ratio > 1), polymorphonuclear percentage (>80%), and synovial CRP (>6.9 mg/L)), a final aggregate score ≥6 indicates an infected patient, while between 2 and 5 indicates the inclusion of intraoperative findings for confirming or refuting the diagnosis [104].

The specific infection of the knee prosthesis begins with the adhesion of bacteria and biofilms’ formation [105,106]. The implant is quickly covered with adhesins (e.g., fibronectin, fibrin, fibrinogen) in the extracellular fluid. Fibronectin is important in the adhesion of *S. aureus* as it can bind proteins that promote bacterial adhesion [107]. Besides the well-known role of fibronectin-binding proteins (*FnBPA* and *FnBPB*), staphylococci harbor a specific locus called “intercellular adhesin locus (*ica*)” that expresses the polysaccharide intercellular adhesion (PIA), one of the fundamental constituents of biofilm [108]. After the initial adhesion, a thin layer of slime produced by the host induces an inflammatory reaction in the host itself [109]. Biofilm formation is the defense mechanism by which these organisms can escape the immune system [110]. As a result, *S. aureus* can invade and colonize immune cells, such as macrophages and neutrophils [110]. Furthermore, bacteria can naturally mutate some essential metabolic genes, giving rise to different subpopulations called small colony variants (SCVs). These mutants are not particularly virulent but can persist viable inside host cells by attenuating the virulence and antibiotic resistance profile [111,112].

### 5.3. Post-Traumatic Osteomyelitis

“Post-traumatic osteomyelitis” means bone infection following an open fracture or following the treatment of a closed fracture with intramedullary nailing or plating to stabilize the fracture [101]. Transcutaneous bacterial contaminations in open fractures are frequent. When bone tissue is involved, bacteria induce an acute inflammatory reaction, as we have seen previously. Bacteria and inflammation spread within the bone and percolate through the Havers systems and periosteum, compromising callus formation [100,113,114].

## 6. Animal Models of Osteomyelitis Overview

### 6.1. The History of Animals as Models of Osteomyelitis

The scientific literature offers numerous reports about AMs to investigate the pathogenesis, the diagnosis, and the treatment of osteomyelitis. In this context, AM’s use is fundamental for developing preventive and effective therapies [115]. In some cases, HO’s clinical manifestation results in extreme variability between patients due to multiple variables (e.g., age, sex, route of infection, anatomical location, and concomitant diseases) that could alter clinical studies. On the other hand, animal models may be used to control and test these factors [115].

Between the second half of 1800 to 1970, the availability of scientific reports targeting animals as models for studying osteomyelitis was minimal. However, at the end of the twentieth century, a progressive increase in the number of reports focused on this topic was noticed, corroborating the hypothesis that the development of antibiotics positively influenced osteomyelitis studies involving AM [116].

The two most pioneering scientific reports about AM (rabbit) of osteomyelitis were done by Rodet (1885) and Lexer (1894), which administered *S. aureus* intravenously to induce the formation of abscesses [117,118]. After several years, three independent research papers: Starr (1922), Haldeman (1934), and Thompson and Dubos (1938), observed that after intravenous and/or intra-bones injection of *S. aureus* in rabbits, typical osteomyelitis lesions microscopically resemble those found in humans [119,120,121]. In all these studies, a common factor was found; the direct injection of living or attenuated bacteria caused the death of animals within a few days after treatment by disseminating abscesses in different organs and with occasional bone lesions [115]. In 1941, Scheman proposed a new rabbit model of osteomyelitis based on *S. aureus* and morrhuate sodium injection directly into the tibial metaphysis. In this study, experimental subjects did not die of sepsis, and chronic osteomyelitis lesions arose within the injection site over several weeks [122].

From these fundamental studies, historically considered the backbone of AM research on osteomyelitis, researchers have developed new models and protocols to formulate preventive approaches to understanding specific molecular features of this clinical condition [115,116]. From the beginning of the twentieth century, the rabbit was not considered the unique model anymore to characterize osteomyelitis, and several other species were further investigated: Hamblen (1968) and Zak (1982) introduced rat models [123,124], Ueno (1974) used mouse models [125], Deysine (1976) and Fitzgerald (1983) focused on dog models [126,127], Passl (1979) worked on acute osteomyelitis in guinea pigs [128], Emslie (1983) studied the first avian (chickens) model of acute HO [129,130], Patterson (1993) developed a mini swine model of chronic mandibular osteomyelitis [131], and Curtis (1995) and Kaarsemaker (1997) worked on an open fracture goat model and a new model of chronic osteomyelitis in sheep, respectively [132,133].

### 6.2. What Should Be Considered When Designing Animal Models of Osteomyelitis?

The complex dynamics that surround osteomyelitis should become simpler when using animal models. However, choosing the best model is one of the most critical decisions. Multiple variables simultaneously act during an experiment involving AM, and each of them should be responsible for misleading and biased during procedures. The most significant variables related to experimental designs are animal species, age of the experimental animal, route of inoculation, bacterial species, infection promoters, and evaluation [115,116]. Table 1 reports the advantages and disadvantages of animal models used to mimic human osteomyelitis.

The main advantages and disadvantages of animal models of human osteomyelitis need to be carefully checked when planning an in vivo experiment. Each model can be used to mimic and elicit specific characteristics of the human disease. For example, large animals (dog, porcine, sheep, and goat) present some anatomical features that can resemble humans, allowing to test new surgical approaches and new therapeutical strategies. On the other hand, the presence of very characterized mouse strains and the development of specific antibodies can be very useful in studying the pathophysiology of the disease using targeted immunohistochemistry or in vivo imaging techniques.

### 6.3. Which Is the Most Appropriate Animal Model for the Experimental Procedure?

It is utopic to talk about the perfect animal species that should mimic osteomyelitis; however, it is possible to define an ideal animal species to understand this disease better. This hypothetical model should have molecular, cellular, structural, and mechanical features found adequately in human bone, together with easy housing and handling, low cost, high tolerance to pharmacological treatment (e.g., antibiotic administration), and a size that allows surgeries that resemble clinical practice [115]. It is clear that each animal model has specific features that make it better for a particular purpose; it is also evident that an in-depth analysis should help establish the best model for each experimental procedure. For example, comparing femoral cortical and lumbar trabecular bone tissues between animals (e.g., dogs, pigs, chickens, and rats) and humans was shown that canine and porcine models have mineral proportions similar to those of humans [140]. Furthermore, pigs and dogs show advantages in refining surgical procedures (e.g., excision of abscesses and implant of prostheses/medical devices) due to the long bone size [141].

On the contrary, the small size of mice, rats, rabbits, and chickens makes them inadequate for long bone surgery procedures [115]. Beyond this, mice, rats, and rabbits’ bones are unique for quantitative microbiology tests (e.g., cell count). In this case, the different gastrointestinal physiologies of these animals can affect the results. For example, rabbits have a pseudoruminant gastrointestinal system, which is unsuitable for testing antibiotics absorption and deposition in long bones [142]. In contrast, mice and rats tolerated broad-spectrum antibiotics [143].

### 6.4. Age of the Animals and Route of Inoculation

The second most crucial parameter about the eligibility of the best animal model for HO studies in long bones is age since this disease arises during childhood [46]. Unfortunately, the importance of this parameter is often underestimated, and the majority of reports do not mention in detail the age of the used models, often referred to as “adult” or “young” animals [115]. In 1943, Weaver and Tayler studied the difference in HO development between young (6–8 weeks old) and adult rabbits [144]. They found that young models were best suited for HO development while, in adult animals, the procedure underwent failure. Another problem relates to sex and sexual development; most studies involving mice used females of 8- to 10-weeks-old [115]. At this stage, mice are sexually active, and this physiological condition was thought to influence the inability to establish large macroscopic lesions in most of these models [145,146,147]. Pigs and avian HO models were developed in growing animals, demonstrating typical lesions commonly found in infants [116].

Three inoculation routes have been commonly used for experimental HO: inoculation into the bone cavity, local inoculation through nutrient arteries (intra-arterial inoculation), and systemic inoculation through intravenous injection [115,116].

**Figure 1:**
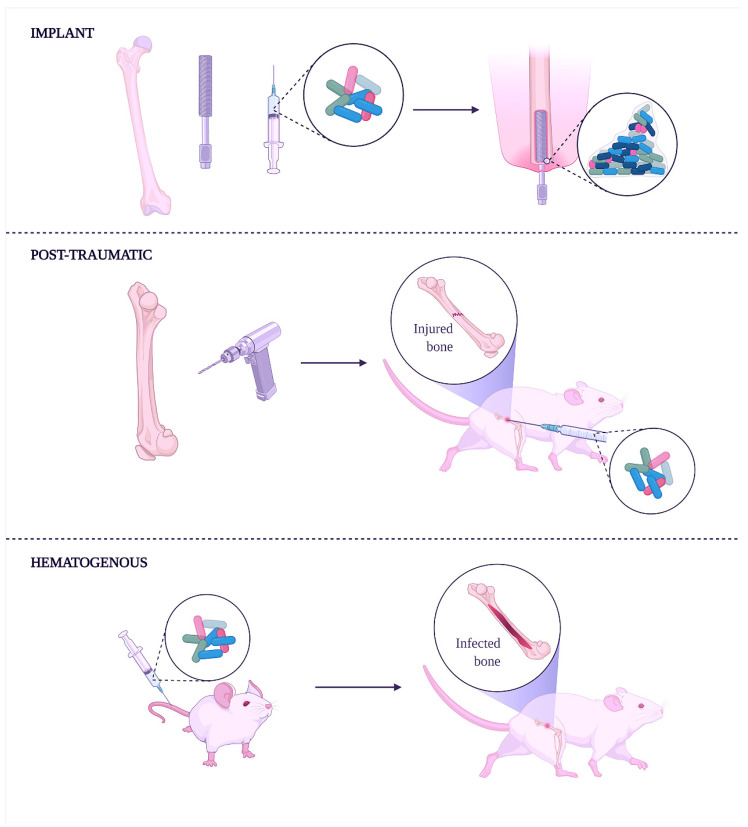
Experimental induction of human osteomyelitis in animal models.

To mimic the HO infection development and microenvironmental alterations in bone tissue is essential to correctly deliver the pathogens in a defined anatomical compartment without creating sepsis or metastatic infection. Intravenous injection results are the least likely to overcome this challenge [148]. Since HO often occurs in a single bone without disseminating systemically, the intra-arterial route is considered the best inoculation to generate a predictable and anatomically confined infection. Moreover, from a surgical perspective, this type of inoculation made in the femoral artery is more manageable than other arteries because of its large diameter and easy anatomical access [149,150] (Figure 1).

The implant-related method (top) induces osteomyelitis using an infected implant to study and develop coating or implants with antibacterial properties. This method is convenient during studies focusing on anti-biofilm molecules. Creating a fracture in the bone followed by its infection with bacteria is the post-traumatic method (middle). In this case, the method helps understand how bacteria can spread along with the bone tissue and through the bloodstream. Moreover, external fixation wires impregnated or covered with antibacterial molecules can be done to characterize the shelf-life of new implants better. Finally, the intravenous injection of bacteria can achieve hematogenous osteomyelitis (lower), spontaneously reaching bones.

### 6.5. Bacterial Species Used to Induce Experimental Osteomyelitis

The most common bacterial species used to induce HO in animal models is *S. aureus*. Many scientists used strains from a private bacterial collection (mainly derived from patients with HO), while others used ATCC strains (e.g., *S. aureus* ATCC 49230, originally isolated from a patient with chronic osteomyelitis). However, HO can also be induced by other bacterial species, including *E. coli*, *P. aeruginosa*, and *S. epidermidis*. From a microbiological point of view, HO is traditionally monomicrobial, and the infusion of a single bacterial species can easily mimic the microbiological environment found in humans. On the other hand, CO is usually polymicrobial [151]. To mimic this complex bacterial environment, the type of strain used and the number of bacteria to be inoculated remain the two pivotal factors to be considered [116]. Hidaka (1985) used a combination of *S. aureus* (1 × 10^4^ CFU/mL) and *P. aeruginosa* (1 × 10^5^ CFU/mL) to experimentally induce osteomyelitis in mice, using silk thread inserted into the metaphysis of the tibia [152]. In 1988, Sakaeda elaborated a combined (aerobes-anaerobes) reliable model of polymicrobial osteomyelitis using clinical isolates of *S. epidermidis* and *E. faecalis* as aerobes (1 × 10^5^ CFU/mL) and *B. fragilis* and *B. bivius* as anaerobes (1 × 10^6^ CFU/mL). The microorganisms were loaded in silk threads and inserted into the bone marrow of a rat. [153]. The literature reports a wide range of bacterial inoculum to experimentally induce osteomyelitis, ranging from 10^2^ to 10^10^ CFU/mL [154]. However, some authors demonstrated that a less than 10^4^ CFU/mL concentration resulted in an unsuccessful infection [114,135]. Helbig (2015) developed a rat model (female, six-month-old Sprague–Dawley) of delayed osseous union achieved by intramedullary inoculation of *S. aureus* (10^3^ CFU/mL) [155]. Some authors used higher bacteria loads. Schaer (2012) developed a sheep model to study the antibiofilm properties of a hydrophobic polycationic coating implanted in the tibia using *S. aureus* ATCC 25923 suspensions (10^6^, 10^8^, and 10^10^ CFU/mL) [156]. Bilgili (2015) used a rat model (male, four-month-old Sprague–Dawley) of osseous union inoculating *S. aureus* (10^8^ CFU/mL) [157]. In conclusion, there is no consensus in the literature on a standardized bacterial inoculum to achieve a single and predictable bone infection [154].

In most studies, bacteria were not administered alone but combined with sclerosing agents (e.g., morrhuate sodium solution and arachidonic acid) to facilitate bone infection. Sclerosing agents act on small blood vessels in the bone’s medullary canal, causing sclerosis of vascular tissue and subsequent necrosis. This action causes a decrease in the local host defense system and positively influences the proliferation of bacteria, leading to osteomyelitis [116]. Standard protocols inject 0.1 mL 5% sodium morrhuate solution for tibia or femur models in rabbits [122,158] and 25 µL in rats [159]. Other ways can be utilized to enhance the development of bone infection. Foreign bodies (e.g., bone cement, silicone catheter, metal wires, intramedullary nail, or rods) are preferred to sclerosing agents not only to physically insert a non-self body directly in medullar or bone marrow cavities but also to study the impact of bacterial biofilm on bone tissues and mimic device-related biofilm infections [160].

Macroscopic and microscopic evaluation of experimentally induced osteomyelitis uses fundamental methods, including clinical observation, microbiology, radiography, and histology [116]. After the bacterial inoculation or device-implant, the continuous record of physiological parameters or clinical symptoms (e.g., temperature, lethargy, and weight loss) can easily indicate the progression of localized infection, soft tissue degeneration, or generalized dissemination of pathogen (sepsis). In addition, typical clinical signs (e.g., erythema, abscess, or sinus formation) should help in the final diagnosis that has to be supported by other microscopic methods. Culture-dependent techniques are the most diffused and used, but recent advances in genomics and the rapid progression in culture-independent protocols open a new era in microbiology. Nowadays, the 16S metagenomic profile can easily detect polymicrobial osteomyelitis without the prolonged time and high costs due to classical culture-based microbiology [136,161,162]. Pineda (2009) suggested some radiographic criteria for evaluating the severity of the infection and the use of other diagnostic techniques to help the final diagnosis of osteomyelitis [163].

## 7. Specific Animal Models

In a recent review of animal models for the studies of implant-related infection, Lovati (2016) stated that among all the available and validated animal models, “*rats represent the most used (53%), followed by mice (25%), rabbits (20%) and large animals (2%)*” [114].

### 7.1. Rat Models

Historically, the introduction of the rat as a model to mimic osteomyelitis (in particular internal fixation models of osteomyelitis) has to be acknowledged by the pioneering studies by Scheman (1941), Hamblen (1968), and Zak (1982), which injected in tibial metaphysis a 5% morrhuate sodium solution followed by a suspension of *S. aureus* [122,123,124]. These types of rat tibial models were recognized as the first to assess chronic osteomyelitis and, at the same time, helped scientists to study the administration and delivery of multiple bacteria [164,165]. Further studies demonstrated that foreign bodies (e.g., fibrin glue, bone cement, fibrin foam) could improve the infection rate up to 85% [116]. In 1985, Rissing drilled a cortical canal in the medullary portion of the tibia; the canal was filled with foreign bodies (calcium phosphate granules) [159]. Other models used the intramedullary insertion of titanium Kirschner wire instead of a canal drilled in the tibial metaphysis. Lucke (2003) studied some antibiotic delivery mechanisms after the fixation of a contaminated Kirschner wire (*S. aureus*) in the tibia. Bisland (2006) studied the effect of photodynamic therapy using a bioluminescent strain of biofilm-producer *S. aureus* grown onto Kirschner wires implanted in the tibial medullar cavity of Sprague–Dawley rats [166,167]. Akiyama (2013) investigated the antibacterial abilities of silver ions (Ag^+^) within hydroxyapatite against an MRSA infection in the medullary canal of rat tibia, demonstrating a time-dependent Ag^+^ release [168].

Beyond the use of rats as the tibial osteomyelitis model, some researchers used this rodent model to recreate femoral implant-associated chronic osteomyelitis. For example, Robinson (2011) developed a model of induced implant-associated osteomyelitis after fracture repair using intramedullary pins followed by inoculation with *S. aureus* (10^4^ CFU/mL) [137]. In the same year, Ozturan studied the efficacy of moxifloxacin compared to teicoplanin in the treatment of a femoral model of osteomyelitis; the results demonstrated that moxifloxacin therapy was an effective alternative to teicoplanin [169].

Two old studies reported the design of a novel rat model for mandibular osteomyelitis (in one case HO); the first one was made in Russia by Solov’ev (1992), followed by the Swedish report of Hienz (1995). Solov’ev drilled the rat mandible and filled the empty cavity with bacteria, following the disease [170]. Hienz injected a 5% sodium morrhuate solution directly into the mandible and tibia, followed by an intravenous injection of *S. aureus* into the femoral vein to mimic hematogenous spread, effectively generating both mandibular and hematogenous osteomyelitis [171]. However, currently, the literature has few reports about rats’ use as a model for mandibular osteomyelitis.

Model of total joint arthroplasty cannot be realized using rats mainly due to the small size of this rodent [165]. Kalteis (2006) contaminated the femoral cavity of rats with *S. aureus* ATCC 29213 and implanted a metal device together with two antibiotics (moxifloxacin or vancomycin). Results indicated that moxifloxacin was more effective than vancomycin [172]. Harrasser (2016) evaluated the effect of a titanium screw coated with hydroxyapatite alone or associated with a low concentration of silver against low and high doses of *S. aureus* ATCC 25923 introduced in the tibial metaphysis of rats. Infections resulted independently using both the bacterial concentrations even in hydroxyapatite alone or associated with a low concentration of silver [173]. In 2013, Haenle generated a tibial defect implanted with a titanium screw and contaminated with low and high inocula of *S. aureus* ATCC 25923 to verify a relationship between bacterial dose and severity of implant-associated infection. Results indicated a significantly high viable bacterial count in bone infected with a high bacterial dose [174]. Fan describes a joint replacement model in the rat with ultra-high molecular weight polyethylene and titanium components. A 3 mm deep hole was drilled in the tibia and immediately injected with *S. aureus* ATCC 12600, and a titanium screw was implanted. The results confirmed the development of a local infection for four weeks [175].

Beyond the well-recognized role of *S. aureus* in inducing experimental osteomyelitis, some researchers used other bacterial strains. [114]. In 1990, Nelson studied an antibiotic-resistant experimental rat model of *Pseudomonas* osteomyelitis without the injection of sclerosing agents, obtaining a reliable model of chronic osteomyelitis [176]. In 2001, Hendricks developed a rat model of polymicrobial osteomyelitis using *S. aureus* and *P. aeruginosa* injected at different concentrations (10^2^, 10^3^, and 10^4^ CFU/mL) into the spinous process lumbar vertebra of Sprague–Dawley rats. Results indicated the synergy between the two bacterial species when low levels of each organism were present in the wound [177]. In 2006, Mecikoglu developed a rat model of periprosthetic infection using *S. epidermidis* ATCC 35984 and Kirschner wire fixed with bone cement into the medullary femoral canal to investigate the eradication of a biofilm-related infection using a proteolytic enzyme (serratiopeptidase). The authors concluded that the enzyme helped bacterial eradication [178]. In 2016, Lovati developed a rat model of *S. epidermidis*-induced non-union of the femoral fracture using different concentrations (10^3^, 10^5^, and 10^8^ CFU/mL) of a biofilm-producing methicillin-resistant *S. epidermidis* injected within fractures, following synthesis with stainless steel plates and screws. A dose-dependent effect between the bacterial inoculum and the non-union rate was demonstrated. Other studies involving rats as models of *S. aureus* bone infection after osteotomy, periosteal reaction, thickening of the cortex, myeloid hyperplasia, and polymorphonuclear cells in granulation tissue have been observed [114,179] (Table 2).

Rat’s bones are sufficiently sized to reproduce fracture patterns and allow drilling and fixation using Kirschner wire and intramedullary nailing. In addition, the medullary canal is large enough to ensure the insertion of foreign bodies to simulate osteomyelitis and allow the implant of orthopedic devices. Compared to large animal models (e.g., sheep and goats), rats are inexpensive and easy to house and maintain for prolonged experimental periods.

### 7.2. Mouse Models

In 1974, Ueno reported the first mouse tibial model. Experimental osteomyelitis was induced by creating a canal into the tibial metaphysis of mice, which was successively filled with silk thread loaded with suspensions of *S. aureus* or *P. aeruginosa* [125]. Some years later, Kobayakawa (1979) used *S. aureus* to create a HO mouse model by inserting a silk thread into the proximal tibial metaphysis. Results demonstrated that when a foreign body was not used, *S. aureus* started to diffuse into most bones and later colonized the femur and tibia, while the foreign body promoted a localized infection [138]. In 1998, Pesanti and Lorenzo worked on Ueno’s same model, using a silk thread with a low dose of *S. aureus* ATCC 49230 directly introduced within the tibial canal of animals. The study aimed to evaluate the activity of IL-4 in inhibiting the function of the osteoclasts during the bone repair process in chronic osteomyelitis [139]. An acute HO mouse model was developed in 1999 by Chandha by an epiphyseal fracture in the proximal tibia, followed by intravenous inoculation of *S. aureus*. The infected mice increased circulating B lymphocytes and CD4^+^ T lymphocytes. Histopathological findings confirmed that polymorphonuclear leukocytes infiltrated the proximal tibia [145]. In 2014, Lovati developed an implant-related infection in type I diabetic mice. A stainless-steel needle was covered with 10^3^ CFU of *S. aureus* ATCC 25923 and inserted within the femoral canal of animals. Prostaglandin E1 (PGE_1_) was systemically injected as a preventive osteomyelitis agent. Results indicated that the simultaneous administration of a PGE_1_ enhanced the local blood flow and improved antibiotic therapy [180]. In mice models, bioluminescent bacterial strains are used to perform real-time tracking of pathogens through bone tissue of host immune cells. In 2008, Li described an implant-associated osteomyelitis murine model using a bioluminescent *S. aureus* strain (Xen29). A stainless steel pin was coated with the pathogen and implanted transcortically through the tibial metaphysis of mice. Serology found a progressive increase in IgM (protective humoral response) after one week, which converts to a specific IgG2b response by day 11 post-infection [181]. In 2010, Bernthal used bioluminescent *S. aureus* and genetically engineered mice expressing fluorescent neutrophils to develop a dose-related model of post-arthroplasty infection. After the infection of the femoral canal with bioluminescent *S. aureus* (ALC2906 strain), both bacteria and host neutrophils (engulfed by phagocyted pathogens) were monitored in real-time using an in situ vivo imaging technique. The result demonstrated the establishment of chronic osteomyelitis with a low grade of inoculum (5 × 10^2^ CFU/mL) [182]. The osteomyelitis mouse model proposed in 2012 by Funao was the first that used in vivo bioluminescence imaging (BLI) without sacrificing mice enrolled. After the femur’s surgical exposition, *S. aureus* Xen29 was inoculated into the medullary cavity. The authors concluded that a high proportion of granulocytes was detected in the infected group’s peripheral blood after seven days. Moreover, serological analyses showed high levels of IL-1β, IL-6, and C-reactive protein [183]. Pribaz (2012) compared the biofilm formation of two bioluminescent *S. aureus* strains (Xen36 and Xen40) on both stainless steel and titanium K-wires implanted within the femoral canal of mice [184]. In 2002, Yoshi developed a mouse model of tibial osteomyelitis and determined, over time concentration of cytokine levels (IL-1β, IL-4, IL-6, and TNF-α) in bone. After the surgical exposition of the proximal tibia, a hole was drilled, and a silk suture (seeded *S. aureus*) was inserted. Results indicated an immediate release (early phase) of IL-1 β and IL-6 due to bone damage (which dramatically decreases after a few days), followed by a rapid production (latent phase) of IL-4 and TNF-α associated with histopathological changes (bone resorption and formation) [185]. In two studies focused on the periprosthetic joint infection through titanium K-wires in mice, Heim (2014, 2015) studied the role of myeloid-derived suppressor cells (MDSC) and their pro-inflammatory activity associated with a bioluminescent and biofilm producer *S. aureus*. These two publications clarified the role of IL-12 in recruiting MDSC and impairing the phagocyte activity, reducing the pro-inflammatory events [186,187] (Table 3).

One of the essential advantages of using mice as models to mimic osteomyelitis is using bioimaging to deeply understand the intimate connection between immune response and pathogen dissemination without euthanizing the experimental group. Beyond this, the small size of this model could be positive to the reduced cost and easy handling, but on the other hand, it makes two-stage surgical revisions and multiple procedures in a single mouse more challenging.

### 7.3. Rabbit Models

In 1885, the first attempt to create an animal model to study osteomyelitis was performed with rabbits [118]. However, only several tries later, the original tibial model with chronic, progressive osteomyelitis in rabbits was described by Scheman in 1941 and then later enhanced by Norden and Kennedy in 1970. In this model, a sclerosing agent (5% sodium morrhuate) was inoculated in the tibia, followed by *S. aureus* to produce chronic osteomyelitis [122,158]. In 1973, Andriole created a model that maintained chronic staphylococcal osteomyelitis for a long period and introduced the possibility of using a foreign body (e.g., stainless steel pin). They fractured the rabbit’s tibia using a simple three-pronged clamp and a stainless steel pin for fixation and were able to follow the infection for up to 18 months, and 88% of the rabbits with bacterial inoculation pinning presented evidence of chronic osteomyelitis [190]. These models relied on the virulence of *Staphylococcus* to create a model of the human disease and demonstrated that an extraneous substance or trauma was necessary to create a chronic, progressive infection. Later on, other authors used the same techniques without fracture [191,192].

When it comes to open fracture rabbit models, the first was published in 1982 by Ashhurst, primarily using the tibia [193]. Worlock then modified this model to generate a model with fixation of an intramedullary rod [194], and since then has been used by other studies [195]. Unfortunately, the intramedullary rod described by Worlock lacks rotational stability.

In 1991, Johansson developed the first model with anaerobic organisms. In this study, Ringer solution was inoculated in each animal’s left femur, while in the right femur, after treating the proximal metaphysis with 5% sodium morrhuate, *Bacteroides fragilis* was inoculated. As a result, all animals developed osteomyelitis on both sides, but with more prominent symptoms on the right one [196].

Rabbit models created to study osteomyelitis show the importance of preparing the bone before inoculation for chronic, progressive infection. Nowadays, several investigators, like Schulz, use these resources to develop methods of applying local therapies to avoid complications of systemic antibiotics and improve efficacy [197].

Rabbits are the smallest animals that can be used as models for prosthesis-related osteomyelitis. In addition, rabbit models of knee arthroplasty components can be found as a model for joint replacements for humans [198], and in 2005, Craig developed a new arthroplasty model in which a metal screw and ultra-high molecular weight polyethylene washer were held to the non-articulating surface of the lateral femoral condyle [199].

The first hematogenous model involving trauma to the proximal epiphysis of the tibia was produced using a three-point bending force over the proximal part of the tibia, creating a reproducible shearing injury to the physis. This was followed by an intravenous injection of a high bacterial load and provided good histologic results [200,201]. In the 1990s, this model was revised by Johansson. Metallic hardware was implemented in the distal tibia without any fracture created before the fixation. After the incision had, *S. aureus* was inoculated into the auricular vein. Unfortunately, this model had a relatively poor infection and high mortality rates [202]. This could be due to the injury that creates a susceptible area to the host immune system, theoretically allowing greater and different infection rates (Table 4).

Nowadays, rabbits are used in many studies involving osteomyelitis because they are relatively inexpensive and because their size makes them versatile, being relatively easy to handle, manipulate, and maintain.

### 7.4. Poultry Models

The poultry model for osteomyelitis was developed to mimic the natural course of acute hematogenous osteomyelitis, often due to traumatic events in the bones and surrounding tissues. The model proposed by Emslie and Nade used *S. aureus* inoculated in chickens. The animals were sacrificed at different time points (6, 12, 24, 48, 96, 192 h). Lungs, liver, and kidneys showed no bacterial infections. The growth rate of animals in the *S. aureus* group compared to the control group significantly decreased. The average weight of the animals after 8 h post-injection decreased in the treated group, while it increased in the control group. The lesions were appreciated even after 12 h post-inoculation. Normal vascularization and supporting mesenchymal cells were missing around the bacterial foci. Inflammatory cells were present in the extravascular spaces and the *lacunae* of chondrocytes surrounding an abscess with necrotic areas. This model confirmed that the bacteria initially reach the metaphyseal vessels, and the occasional involvement of the epiphysis was analogous to what occurs in human infants. In addition, while the cartilage matrix prevents one further spread of bacteria, it creates a protective barrier that prevents access to inflammatory cells [129]. In 1990, Daum and colleagues developed a chicken model of staphylococcal bacteremia, septic arthritis, and osteomyelitis. Chickens were intravenously injected with three different *S. aureus* loads, developing bacteremia in 80%, 90%, and 100% of the treated groups of animals, respectively. Osteomyelitis was developed 1 up to 23 h after inoculation [204].

Further, researchers designed experimental avian models to develop osteomyelitis after intravenous injection of appropriate amount and strains of *Staphylococcus* spp. to induce bacteremia without triggering septicemia [205,206,207]. Researchers studied the role of double-stranded RNA (dsRNA) in the infected bone of a *Staphylococcus*-induced chicken osteomyelitis model at a molecular level. They showed that dsRNA accumulation in bone tissues activated NACHT, LRR, and PYD domain-containing protein (NLRP)3 inflammasome and increased IL18 in vivo and in vitro, thus identifying dsRNA as a new target for the treatment of osteomyelitis [208] (Table 5).

### 7.5. Large Animal Models

#### 7.5.1. Canine Models

In orthopedic research, dogs are recognized as one of the most suitable models due to their high similarity (compared with all other non-human species) with human bones in density and mineral composition [140].

In 1976, Deysine et al. proposed a canine model of acute hematogenous osteomyelitis, which involved the injection of barium sulfate with 10^5^ CFU/mL of *S. aureus* into the tibial nutrient artery. Unfortunately, the induced lesions differed from the human ones [127,164]. Consequently, in 1983, Fitzgerald proposed a canine model of chronic osteomyelitis due to open fracture. In this case, *S. aureus* load was inserted into the dog’s tibia within artificial support consisting of acrylic bone cement. The resulting infection was compatible with subacute osteomyelitis or infection following total knee arthroplasty. This model is useful for studying surgical procedures, evaluating the effectiveness of therapies, and investigating reactions from foreign bodies [126]. In 1985, Petty et al., using 187 dogs, evaluated the effects of different implant materials on the infection rate after bacterial inoculation. At the femur level, a suspension of bacteria was inserted (*S. epidermidis*, *S. aureus*, and *E. coli*), and then the different implants were placed. They saw that any material used increased the risk of *S. aureus* infection, while *E. coli* and *S. epidermidis* infections were successfully induced using polymethylmethacrylate (PMMA) polymerized in vivo [209]. In 1994, Garvin et al. used the model proposed by Fitzgerald to study gentamicin’s effect on two different implants. After six weeks of treatment, the animals were sacrificed. The PMMA implant was removed intact, while only fragments of the polylactide/polyglycolide were extracted (this was due to reabsorption). Local therapy had better effects than systemic therapy. The reabsorption of the biodegradable implant suggested a good strategy to avoid removing the implant after therapy [164,210].

However, despite the positive anatomical attributes shown by these models, nowadays, few canine models are still developed and used, mostly due to ethical concerns and economic reasons.

#### 7.5.2. Small Ruminants’ Models (Sheep and Goat)

One of the most desirable characteristics of sheep that made them eligible for use as models is the size of their bones, which allow for mimicking long bone osteomyelitis; moreover, the rate of osteogenesis between humans and sheep remains similar. However, two microscopic differences make sheep not eligible for microenvironment studies; the sheep bones are denser and present fewer Harversian canals [140,211].

In 1997, Kaarsemaker et al. developed a sheep chronic osteomyelitis model for toxicological and therapeutic studies. A sclerotic agent and *S. aureus* were injected into the medullary cavity of the tibia. Chronic inflammation, osteolysis, new bone tissue, bacteria, and granulation tissue developed. Unlike canine models, using the sclerosing agent instead of an implant made this an excellent model for studying the factors influencing surgical therapies and drug release systems [132,164]. More recently, an intramedullary nailing contaminated fracture sheep model was developed. After the exposition of the tibia, an osteotomy was created. An inoculum of *S. aureus* was loaded on bovine type I collagen. Researchers concluded that nail fixation might not be appropriate in all models because it could increase bacterial virulence, causing less efficacy of antibiotic treatment [212].

Unlike the anatomical similarities with sheep, goats’ models have not been widely used and developed. However, like sheep, these animals can easily mimic human long bones osteomyelitis.

In 2005, Salgado et al. developed a model of chronic osteomyelitis. A hole was drilled, and sodium morrhuate and *S. aureus* were injected. Ultimately, 96% of the animals highlighted the typical pathology findings of osteomyelitis, both concerning radiographic and histological analysis [164,213]. Similar to the study by Salgado, Beardmore created a similar defect model by drilling a hole in the proximal tibial metaphysis of goats and filling it with *S. aureus*. Without using a sclerosing agent, osteomyelitis was induced [214] (Table 6).

#### 7.5.3. Porcine Models

The pulmonary intravascular macrophages, which decrease bacteremia and enhance survival following hematogenous injection of bacteria, are an essential feature that makes pigs the model of choice for HO procedures [218].

Jensen and colleagues used a stainless steel implant to mimic implant-associated tibial osteomyelitis. A K-wire was drilled in the medullary cavity of the tibia of SPF pigs (specific pathogen-free), and *S. aureus* was loaded in the artificial cavity with a small steel implant. Animals were monitored for five days, and all groups noted signs of localized, acute osteomyelitis to varying degrees on computed tomography (CT) scan and implant cavity cultures [219].

A porcine model of post-traumatic osteomyelitis was described in 2001 by Hill. A steel fragment was fired on the right tibia of pigs to mimic a ballistic injury; the wound was contaminated with *S. aureus* ATCC 29213 loaded on a strip of sterile bovine collagen placed into the bone defect. All animals developed osteomyelitis and infection of surrounding soft tissues. After 14 days, a radiographic examination showed a central radiolucent area and surrounding sclerosis. Histology examination confirmed the diagnosis of osteomyelitis after finding purulent material in bone and associated osteonecrosis [220].

In 2010, Jensen and colleagues developed a hematogenous porcine-based model. A catheter was placed in the left ear vein to inoculate *S. aureus* strain at different time points. In animals euthanized at 12, 24, and 48 h, long bones and lungs showed clear signs of infection. However, after 48 h, the pulmonary bacterial load diminished, and with no evidence of bacteremia, this was attributed to the pulmonary intravascular macrophages that can phagocyte *S aureus* [142,218]. In 2017, Jensen explored a recent modification of this model. An intra-arterial catheter was placed into the right femoral artery, followed by an *S. aureus* infusion. The pigs were monitored after inoculation and recovery from anesthesia the following days and noted signs of disease [221] (Table 7).

As stated above, the pharmacological approach to treating osteomyelitis resides in systemic antibiotic administration. Since an adequate antibiotic concentration can reach the infection site, the physiology of animal nutrition is essential in choosing a particular model. Pigs, like humans, are omnivores; they respond to antibiotics administration and diffusion similarly, making them eligible for oral and/or systemic antibiotic treatment evaluation [140,219]. Two of the main disadvantages of porcine are their rate of bone growth (faster than humans) and the length of both tibia and fibula, which limit their use in implant evaluation procedures [219].

## 8. Conclusions

Using animal models to mimic and characterize human osteomyelitis remains a fundamental challenge and depends on the physiology and specific anatomical features of the animals used. The selection of the most appropriate model has to be done considering multiple characteristics: the disease under investigation, pros and cons of the specific animal model, type of experimental design, experimental time-points, reliability, and repeatability of the predicted model. Nowadays, used animals have specific advantages and disadvantages mainly derived from handling, costs, housing, dimension, and anatomical similarity to human lesions. For example, rats and mice have been overused since they were introduced as experimental animals. Rabbits and other large animals have adequate dimensions to ensure a more precise surgical intervention, but some are too hard to handle, and results are not repeatable between laboratories. The refinement of procedures, including animal models, the definition of new guidelines for a scientifically acceptable animal-driven experimentation, and the use of complementary technologies could help the scientists choose the most appropriate model and drive the research in finding new strategies to better understand also at molecular levels what happens during the crosstalk between pathogen multiplication, immune system, and bones.

## Figures and Tables

**Table 1:** Pros and cons of using animal models of human osteomyelitis.

Species	Pros [1,2,3,134,135,136,137,138,139]	Cons [1,2,3,134,135,136,137,138,139]
Small models	Evaluation of pathophysiology and novel treatment strategies	Failure in systemic antibiotic treatment evaluation studies due to the physiology of the gastrointestinal tract
	Adaptable to pathological conditions (easy manipulation)	Very small joints–in situ examination is impossible
	Development of well-characterized mouse strains (knock-out or transgenic models)	Limitations associated with existing surgical approaches
	Use of specific and well-known antibodies	Limited or rapid cortical remodeling
	Bone turnover is similar to human	Cortical bone composition (e.g., hydroxyproline and protein content) differs from that of humans
		Biohazard risk related to handling infected animals (infected bites)
		Growth plates never close in mice and rats
		Ethical concerns
Large models	Higher life span	Ethical concerns
	Larger skeletal surfaces allow mimicking internal and external fixation techniques and implants commonly used in humans	High cost (breeding, manipulation)
	Rate of osteogenesis (sheep and goat)	Higher rate of bone growth than humans (porcine)
	High similarity to human bones in density and mineral composition (dog and porcine)	Bones are denser and present fewer Harversian canals (sheep)

**Table 2:** Rat models of orthopedic infections.

Model/Strain	Gender	Age/Weight	Microorganism/ Concentration	Disease Model	Site of Inoculum	Osteomyelitis Induction	Timepoint	Aim of the Study	Results	Ref.
Inbred cr/rar and outbred Sprague–Dawley albino	M	Inbred: 200–400 g; Sprague–Dawley albino rats: 400–500 g	*S. aureus* phage type 52/52A/80. 3.0 × 10^6^ CFU	CO	Tibia (bone marrow)	Sodium morrhuate 5% and arachidonic acid	35 days	To verify if arachidonic acid could facilitate experimental osteomyelitis	Arachidonic acid was a strong facilitator of osteomyelitis.	[159]
Sprague–Dawley albino	M	300–400 g	*P. aeruginosa* (field strain isolated from a patient with osteomyelitis). 2.4, 3.3, 4.8, 6.4, and 7.1 log CFU were used to determine ID50. Log 5.8 CFU was used for experimental inoculation.	CH	Tibial metaphysis		21 days (for ID50 determination) and 63 days for the experiment	To create a rat model of chronic *P. aeruginosa* osteomyelitis that did not require promoting agents	The ID50 was log 4.0 CFU with an ID100 of log 6.4. In the rat model, the establishment of *P. aeruginosa* osteomyelitis does not require promoting agents.	[176]
White	na	na	*S. aureus*	Mandibular osteomyelitis	Drilled cavity in mandibular cavity	na	na	To develop a reliable model of mandibular osteomyelitis	Preclinical study of new drugs and physiotherapeutic methods can be obtained after the used of this model	[170]
Wistar	F	180–220 g	*S. aureus* Phillips (field strains from osteomyelitis). 5 × 10^4^, 5 × 10^6^, 5 × 10^7^, or 5 × 10^8^ CFU (surgically exposed mandible). 5 × 10^6^, 5 × 10^7^, or 5 × 10^8^ CFU (surgically exposed tibia). 5 × 10^6^ and 5 × 10^7^ CFU (control B and control C). 10^8^ CFU (control D).	HO	Intramedullary injection of sodium morrhuate in mandible and tibia. Bacteria were injected into the femoral vein.	Sodium morrhuate 5%	14 days	To establish and evaluate a new rat model of haematogenous osteomyelitis	No pathologic changes were produced in animals undergoing only surgery but receiving sodium morrhuate (control). In the treated group, osteomyelitis was successfully established.	[171]
Sprague–Dawley	M	300 g	*S. aureus* ATCC 29213 or *P. aeruginosa* ATCC 27853. 10^3^ CFU in one group and 10^6^ CFU in the other for *S. aureus* and ascending concentrations for *P. aeruginosa*. 10^3^ CFU of *S. aureus*, 10^3^ CFU of *P. aeruginosa*, or 10^3^ CFU of both *S. aureus* and *P. aeruginosa*.	Complex orthopaedic wounds	Lumbar spinous process	na	14 days	To determine whether synergy exists between *S. aureus* and *P. aeruginosa* in a rat model of complex orthopaedic wounds	When low levels of each organism were present in the wound, synergy existed. The ability of *S. aureus* to cause infections qis enhanced by low concentrations of *P. aeruginosa*.	[177]
Sprague–Dawley	F	5 months	*S. aureus ATCC 49230*. 10^3^ CFU	Implant–related osteomyelitis	Proximal tibia metaphysis (medullary cavity)	Poly (D,L–lactide)–coated Kirschner wire	42 days	To test the efficacy of a new biodegradable, gentamicin–loaded poly(D,L–lactide) coating	The implant–related infection was significantly reduced by PDLLA + 10% gentamicin.	[166]
Sprague–Dawley	F	250–300 g	*S. aureus* Xen29 (genetically engineered using Gram–positive lux transposon plasmid pAUL–Atn 4001 luxABCDE km^r^). 10^6^ CFU/mL. Biofilm–coated K–wire (0.5 cm long).	Implant–related osteomyelitis (biofilm model)	Proximal anterior margin of the tibial epicondyle	Arachidonic acid (50 µg/mL in 0.9% NaCl solution)	10 days	To investigate photodynamic therapy (PDT) as alternative treatment for osteomyelitis using bioluminescence	1 mM of 5–aminolevulinic acid and methylene blue 0.1 mM can mediate the sensitivity of *S. aureus* at 5 J cm^−2^ light dose with ≥4log_10_ cell kill	[167]
Sprague–Dawley	na	417 g	*S. epidermidis* ATCC 35984. 10^5^ CFU/cavity	Implant–related infection	Cortex of the intercondylar notch of the femur	na	14 days (control group), 56 days (treated groups)	To evaluate the effect of serratiopeptidase in the eradication of periarticular hardware	Bacterial growth was reduced in the treated group by serratiopeptidase and antibiotic together compared to animals inoculated with antibiotics alone	[178]
Wistar	M	12–14 weeks, 423–481 g	*S. aureus* ATCC 29213. 10^7^ CFU	Implant–associated infection	Femoral medullary cavity	na	21 days	To assess the antibiotic efficacy of moxifloxacin in implant–associated infections	Animal mortality 0%. The efficacy of moxifloxacin was significantly greater (*p* < 0.01) than that of vancomycin.	[172]
Wistar	na	300–350 g	*S. aureus* ATCC 29213. 10^7^ CFU	Implant–related chronic osteomyelitis	Medullary cavity of femur	na	28 days	To test moxifloxacin compared to teicoplanin in chronic implant–related osteomyelitis	For moxifloxacin–group compared to teicoplanin–group the decrease of bacterial counts was more prominent (*p* = 0.001).	[169]
Sprague–Dawley	M	250–300 g	*S. aureus* (field strain isolated from a patient with an infected total hip arthroplasty). 10^4^ CFU	Femur fracture model	Medullary cavity of femur	na	21 days	To develop a model of induced implant–associated osteomyelitis following fracture repair	Between the control and *S. aureus* group, by one week after surgery/inoculation, significant differences in the radiographic score for osteomyelitis were detected.	[137]
Sprague–Dawley	M	10–w, 283–401 g	Methicillin–resistant *S. aureus* (field strain isolated from a patient with septicemia). 1.0 × 10^2^ CFU	Implant–related osteomyelitis	Tibial medullary cavity	na	28 days	To develop an antibacterial coating with Ag–containing hydroxyapatite (Ag–HA)	Antibacterial activity of Ag–HA coating was shown against MRSA. Serum Ag ion concentrations reached a peak at about 48 h	[168]
Sprague–Dawley	F	na	*S. aureus* ATCC 25923. 10^6^, 10^5^, 10^4^ and 10^3^ CFU	Implant–associated infection	Medial proximal tibial metaphysis	na	42 days	To evaluate a novel animal model for the generation of implant–associated infections in the tibial metaphysis of rats	A higher viable count was observed in peri–implant bone samples from animals inoculated with 106 CFU. However, there could be no correlation between initial load and concentration after sacrifice.	[174]
Sprague–Dawley	M	12 w	*S. aureus* DSM 28763 (field strain isolated from wound infection; genome sequenced, biofilm producer). 10^3^ CFU	Implant–related infection	Tibia	na	42 days	To determine if the prophylactic administration of TLR9 ligand CpG ODN type B would affect a model of implant–related chronic infection	Results indicated that the bacterial load in the infected tibia was reduced at the beginning of infection but failed to prevent the development of chronic infection.	[179]
Wistar	M	12 weeks, 300–350 g	Methicillin–resistant *S. epidermidis strain GOI1153754–03–14* (field strain from infected knee prosthesis). 1 × 10^3^, 1 × 10^5^ and 1 × 10^8^ CFU/rat	Fracture model	Non–critical midshaft full–thickness defect in femur	na	56 days	To understand the role of subclinical bacterial contaminations in the non–union development	Bone healing was prevented in low–grade *S. epidermidis* contamination. Bacterial inoculum and non–union rate followed a dose–dependent relation	[114]
Wistar	M	5 months, 353–401 g	*S. aureus* ATCC25923. 10^2^ CFU (Group I–IIA), 10^3^ CFU (Group I–IIB)	Implant–related osteomyelitis	Proximal lateral tibial metaphysis	na	42 days	To evaluate a low bacterial inocula animal model of tibial metaphysis and investigate osseointegration of the implants coated with hydroxyapatite (HA) and low–dosed HA–silver (HA–Ag)	No systemic infection registered. Infection was induced, independently whether bacterial load used and implant inserted.	[173]
Sprague–Dawley	M	350–400 g	*S. aureus* Xen 29 ATCC 12600. 2 × 10^7^ CFU (injected in 8 mm length–hole), 8 × 10^7^ CFU (injected into the joints)	Periprosthetic joint infection	Lateral femoral condyle	na	28 days	To develop a joint replacement model with ultrahigh molecular weight polyethylene (UHMWPE) and titanium components	Clinical infection indicators such as osteolysis, loosening of the implants were observed for 4 weeks	[175]

Abbreviation: na = not available

**Table 3:** Mouse models of orthopedic infections.

Model/Strain	Gender	Age/Weight	Microorganism/Concentration	Disease Model	Site of Inoculum	Timepoint	Aim of the Study	Results	Ref.
CD1	na	>6 months	*S. aureus* ATTC 49230. 2 to 3 mm length of 4–0 suture seeded with *S. aureus*.	Chronic osteomyelitis	Proximal tibia	2 h–28 days	To investigate the role of interleukin 4 in osteoclast activation and development of chronic osteomyelitis	IL4 may help to block the osteoclast reaction, which leads to more bone loss.	[139]
C3H/HeJ	na	8–10 weeks; 20–25 g	*S. aureus* LS-1. 5 × 10^7^ UFC (injected into the tail vein).	Acute hematogenous osteomyelitis	Tibia (incomplete cartilaginous fracture)	7 days	To study the immunological responses to *S. aureus* bone infection	An increase of splenic B lymphocytes and in lymph–node CD4+ T lymphocytes was observed.	[145]
ICR	F	5–weeks; 25 g	*S. aureus* E-31461. 4.6 × 10^5^ CFU/suture	Tibial osteomyelitis	Tibia	28 days	To evaluate local levels of IL-1 β, IL–4, IL-6, and TNF–α, in a model of murine osteomyelitis due to *S. aureus*	Levels of IL-1β and IL-6 in infected bone were elevated in the early post–infection period and then decreased. TNF levels remained elevated 3 to 28 days post–infection, while IL–4 levels were elevated late in the infection.	[185]
C57BL/6	F	6–8 weeks	*S. aureus* UAMS-1 ATCC 49230 and *S. aureus* Xen29 (derived from ATCC 12600). 9.5 ± 3.7 × 10^5^ CFU/pin of UAMS-1 and 4.2 ± 0.5 × 10^5^ CFU/pin of Xen29.	Implant–associated osteomyelitis	Tibial metaphysis	18 days	To develop a novel murine model of implant–associated osteomyelitis using steel pin coated with *S. aureus*	Histology confirmed all the characteristics of the associated implant. After one week, the mice produced IgM, which converted to IgG 11 days after implantation.	[181]
C57BL/6 wildtype and LysEGFP	M	12–weeks	*S. aureus* SH1000 strain, ALC2906 (contains the shuttle plasmid pSK236 with the penicillin–binding protein 2 promoter fused to the luxABCDE reporter cassette). 5 × 10^2^, 5 × 10^3^ and 5 × 10^4^ CFU/mouse	Post–arthroplasty infections	Knee joint	7 and 14 days	To develop a model of post–arthroplasty Infection combining the use of bioluminescent bacteria and genetically engineered mice that possess fluorescent neutrophils (LysEGFP mice)	Chronic osteomyelitis was developed in mice infected with a low bacterial load, while acute osteomyelitis was developed in those who received 103 and 104. In vivo bioluminescence EGFP–neutrophil signals and fluorescence of LysEGFP mice are highly correlated with Ex vivo bacterial counts.	[182]
BALB/c	M	12 weeks; 20–25 g	*S. aureus* Xen-29. 1.0 × 10^8^ CFU	Osteomyelitis	Femur	Not necessary	To establish a real–time quantitative mouse model of osteomyelitis using bioluminescence imaging	In infected mice, serum levels of interleukin–6, interleukin–1β and C–reactive protein were significantly higher.	[183]
C57BL/6 and LysEGFP	M	12–weeks	*S. aureus* ALC2906 *S. aureus* Xen29 (derived from the pleural fluid isolate ATCC 12600 with an antibiotic marker), *S. aureus* Xen40 (derived from the osteomyelitis isolate UAMS–1, inside chromosome) and *S. aureus* Xen36 (derived from the bacteremia isolate ATCC 49525, integrated into a stable plasmid). 1 × 10^2^, 1 × 10^3^ and 1 × 10^4^ CFU.	Post–arthroplasty infections	Femur	42 days	To study the pathogenesis of post–arthroplasty infections with the use of bioimaging and non–invasive technology	A chronic post–arthroplasty infection model was developed. Up until day 10 ALC2906 had an increase in bioluminescent signals. On day 42, biofilms were detected on the implants inoculated with ALC2906. These results suggest that the construct was lost during in vivo replication.	[184]
NOD/ShiLtJ	F	23.3 ± 1.3 g	*S. aureus*. 10^3^ CFU/mouse	Implant related infection	Femoral canal	28 days	To investigate the effect of a PGE_1_ vasodilator on the incidence of surgical infections in diabetic mice	Limited signs of infection were identified in mice treated with the combination of a PGE1 and an antibiotic using micro–CT and histological analysis.	[180]
C57BL/6	M	8 weeks	*S. aureus* USA300 LAC. 10^3^ CFU	Orthopaedic biofilm infection	Femur	28 days	To examine the functional role of Myeloid–derived suppressor cells in shaping the anti–inflammatory milieu during *S. aureus* orthopedic biofilm infection	Increased expression of Arg–1, iNOS and IL-10. Bacterial clearance was improved due to the targeted depletion of MDSC and neutrophils using mAb 1A8 (antiLy6G).	[186]
C57BL/6NCr	M	8 weeks	*S. aureus* isolate USA300 LAC. 10^3^ CFU	Orthopaedic implant infection	Femur	28 days	To study the pro–inflammatory ability of IL-12 in myeloid–derived suppressor cell recruitment and bacterial persistence	Several cytokines (IL-12p40, IL-1β, TNF–α, and G–CSF) and chemokines (CXCL2, CCL5) were significantly elevated. In both p40 and p35 KO Mice MDSC recruitment was significantly reduced.	[187]
C57BL/6	M	8 weeks	*S. aureus*. 2 × 10^3^ CFU	Implant–associated osteomyelitis	Mid–diaphysis of the femur	3 days	To develop a model of implant–associated *S. aureus* osteomyelitis to study the expression of osteomyelitis associated genes (ERBB2, TWIST1, and NANOG)	Around the infected implant, an upregulation of TWIST1 in macrophages and an accumulation of macrophages was observed. In addition, the expression of TWIST1, MMP9, and MMP13, together with the migration and phagocytosis function of 264.7 cells were increased.	[188]
(NOD)–scid IL2R_γ_^null^ (NSG) mice	F	na	*S. aureus*. 5 × 10^5^ CFU/mL	Orthopaedic implant infection	Tibia	14 days	To study the response of human immune cells during chronic *S. aureus* bone infections engrafting mice with hematopoietic stem cells (huNSG)	Compared to the control group, huNSG mice have increased weight loss, osteolysis, and bacterial spread to internal organs. Moreover, through flow cytometry and immunohistochemistry, more human T cells are present in infected huNGS mice than in uninfected ones.	[189]

Abbreviations: na = not available.

**Table 4:** Rabbit models of orthopedic infections.

Model/Strain	Gender	Age/Weight	Microorganism/Concentration	Disease Model	Site of Inoculum	Osteomyelitis Induction	Timepoint	Aim of the Study	Results	Ref.
Rabbits	na	2–2.25 lb	*S. aureus*	HO and direct inoculation	Metaphysis of the tibia	5% sodium morrhuate	8 weeks	Preliminary report on new method of producing experimental osteomyelitis in rabbits	The authors were able to keep the animals alive for an indefinite period (at least 8 weeks).	[122]
New Zealand white rabbits	na	4–4.5 lb	*S. aureus* phage type 52/52A/80 (field strain isolated from a child with osteomyelitis), *S. aureus* 209 P and *P. mirabilis* (field strain from a urinary–tract infection). 3 × 10^6^ CFU (*S. aureus* phage type 52/52A/80 only).	Chronic osteomyelitis	Tibial medullary cavity	5% sodium morrhuate	60–180 days	To establish a model of chronic osteomyelitis.	The injection of *S. aureus* together with sodium morrhuate could induce osteomyelitis. Animal inoculated with *P. mirabilis* showed the same radiologic lesions observed from those infected with *S. aureus*	[158]
New Zealand white rabbits	F	3–6 kg	*S. aureus*. 2 × 10^4^, 2 × 10^5^, 2 × 10^6^, 2 × 10^7^, 2 × 10^8^, 2 × 10^9^ CFU	Implant–related osteomyelitis	Tibial marrow cavity	na	180 days	A new model of chronic staphylococcal osteomyelitis	The results of this study indicate that chronic staphylococcal osteomyelitis can be produced in the rabbit tibia in the presence of a metallic implant.	[190]
New Zealand white rabbits	M	>3.5 kg	*S. aureus* (phage type 29). 10^5^, 10^6^, 10^7^ CFU	Implant–related osteomyelitis	Tibial medullary cavity	na	8 weeks	To develop a model of induced implant–associated osteomyelitis following fracture repair	The study was successful in developing a model that could be used for other studies in osteomyelitis	[194]
New Zealand white rabbits	na	na	*Bacteroides fragilis*. 10^7^ CFU	Single strain osteomyelitis infection	Medullary cavity of the proximal tibial metaphysis	na	18 weeks	To test a new anaerobic osteomyelitis model	This method gave a high infection rate with reproducible immunologic, roentgenographic, and histologic reactions	[196]
Chinchilla–Bastard rabbits	F	3.25–4.79 kg	*S. aureus*. 3 × 10^5^ CFU	Implant–related osteomyelitis	Proximal end of the femur	0.1 mL 5% sodium morrhuate	6–8 weeks	To find a rabbit model to perform more local therapeutic strategies on the infected bone	The new technique did not influence the motion of the hind limb and mimicked well the intramedullary pinning of long fractured bones, but it did pose some risks for postoperative infections	[197]
New Zealand White rabbits	M	±4.2 kg	MRSA. 0 CFU in one knee and 10^4^ CFU in the contralateral knee (Group A) 10^2^ CFU in one knee and 10^3^ CFU in the contralateral knee (Group B).	Implant–related osteomyelitis	Knee	na	7 days	To design and evaluate a novel small animal model for the investigation of biomaterial centered infection in total joint arthroplasty	This model closely simulates the biologics, and not the mechanics, of human prosthetic knee replacement and is a valuable tool to develop new systemic and local anti–infective strategies	[199]
New Zealand White rabbits	M and F	7–8 months, 2.04 ± 0.09 kg	na	Segmental bone defect in the radial diaphysis	Radius bone	na	30 days	Development of a novel atrophic non–union model in rabbits	The radiographic signs of healing were completely absent in all the rabbits on 30th postoperative day, indicating inert bone ends.	[203]

Abbreviations: na = not available.

**Table 5:** Poultry models of orthopedic infections.

Model/Strain	Gender	Age/Weight	Microorganism/Concentration	Disease Model	Site of Inoculum	Timepoint	Aim of the Study	Results	Ref.
Fowl Rhode Island Red	na	4–6 weeks	*S. pyogenes*; 3 strains: Strain 1641; Strain 8217 phage type 81/+; strain 8272 type 29/7/42E/42D/81. 0–10^8^ CFU	Spondylitis	Intravenous	15–18 days	To describe outbreaks caused by *S. pyogenes* and report the disease’s experimental reproduction.	The condition was not verified on individual birds but on a flock basis; furthermore, did not appear to be related to particular poultry breeds.	[206]
Broiler	M	29–days	*S. aureus* phage type 6/42E/53/77/83A/84. 1 × 10^4^ to 10^8^ CFU/kg	Acute hematogenous osteomyelitis	Wing vein	6, 12, 24, 48, 96, and 192 h	To describe a highly reproducible experimental model of acute hematogenous osteomyelitis in chickens closely mimics the human disease.	Osteomyelitis was produced quite easily. Within the periosteum adjacent to the metaphysis lesions were observed, while in the lungs, liver and kidneys, no bacterial lesions were observed.	[129]
Broiler	M	35–days	*S. aureus*. 10^5^ CFU in one group and 10^7^ CFU in the other.	Acute osteomyelitis	Intravenous	14 days	To record a flock outbreak of femoral head necrosis in broiler chickens due to infection with *S. aureus*.	It has been observed that. *S. aureus* is a pathogen with a tropism for bone growth.	[205]
Broiler	M	30–days	*S. aureus* strain Duntravis, capsular type 8 isolate. 10^5^, 10^6^, or 10^7^ CFU	Osteomyelitis and septic arthritis	Intravenous	14 days	To study the occurrence, magnitude, and kinetics of bacteremia and the resultant osteomyelitis and septic arthritis	From 1 to 23 h after inoculation, osteomyelitis remained uniform in continuously bacteremic animals	[204]

Abbreviations: na = not available.

**Table 6:** Sheep and Goat models of orthopedic infections.

Model/Strain	Gender	Age/Weight	Microorganism/ Concentration	Disease Model	Site of Inoculum	Timepoint	Aim of the Study	Results	Ref.
Goats	M/F	1–4 years, 25–45 kg	*S. aureus* ATCC 700260. 4 × 10^9^ CFU	Chronic osteomyelitis	Tibia	12–16 weeks	To develop a model of tibial osteomyelitis	96% of the animal have radiographic evidence of osteomyelitis. Local osteomyelitis was developed.	[213]
Suffolk–cross breed	F	55–80 kg	*S. aureus* ATCC 29213. 3 × 10^8^ CFU	Fracture	Tibia	3 weeks	To study the outcome of a heavily contaminated fracture	The entire length of the implant induced infection in animals. intramedullary nailing should not be used as a first treatment for heavily contaminated fractures.	[212]
Texel crossbreed sheep	na	3–5 years, 47–64 kg	*S. aureus* PS 8386. 8 × 10^8^ CFU	Chronic osteomyelitis	Tibia (3% tetradecylsodiumsulphate solution used as sclerosing agent)	12 weeks	To develop a large animal model for chronic osteomyelitis	Localized soft tissue swelling, pain during the acute phase, and limping in all sheep were considered clinical signs of infection.	[132]
Spanish goats	na	37–50 kg	*S. aureus* ATCC 29213. 9.42 × 10^4^ CFU	Open fracture	Proximal tibial metaphysis	21 days	To evaluate the prophylactic treatment of bone–graft substitute using locally delivered antimicrobial	The use of tobramycin–impregnated calcium sulfate pellets and demineralized bone matrix prevented intramedullary dissemination of *S. aureus*	[214]
Goat	F	40–50 kg	*S. aureus* ATCC 25923. 2 × 10^4^ CFU	Open fracture	Tibia (intramedullary nails)	5 weeks	To develop a large animal model to study antimicrobial coated bone implants	Intramedullary nails allowed to treat fracture. At 5 weeks, the treated goats lost 7% of initial body weight but was able to ambulate. The control animals were not able to deambulate and lost 8.4% of initial body weight.	[215]
Saanen goats	F	na	*S. epidermidis* HBH276 (field strain isolated from neonate). 3 × 10^5^ CFU	Orthopaedic infection	Tibia	3 weeks	To study the impact of an electric percutaneous current in preventing implant associated infection.	The low amperage electric current prevents infections of percutaneous pins implanted in Tibia	[216]
Dorset–cross ewes	na	2.5–3.5-years	*S. aureus*. 2.5 × 10^6^ CFU	Implant–associated infection	Tibia	3 months	To develop a surface modification of titanium fracture hardware with vancomycin to prevent bacterial colonization in a large animal model	The modified titanium plates treated with antibiotic–derived compounds inhibited the colonization of the implant. Moreover, treated groups showed bone–healing.	[217]

Abbreviations: na = not available.

**Table 7:** Porcine models of orthopedic infections.

Model/Strain	Gender	Age/Weight	Microorganism/Concentration	Disease Model	Site of Inoculum	Timepoint	Aim of the Study	Results	Ref.
Domestic Landrace	na	12 weeks	*S. aureus* (haemolyticus). 2 × 10^8^ CFU	Infectious bone diseases	Femur	16 days	To study the effect of gentamicin embedded in palacos bone cement.	The number of germ populations is reduced significantly by the antibiotic released in a microbiologically active concentration. The number of germs in control group remained at a high level	[222]
Yucatan mini swine	F	2–5 years, 68–95 kg	*S. aureus* ATCC strains 6538P, 25923, and 29213. 10^8^–10^9^ CFU	Chronic Mandibular Osteomyelitis	Mandible (5% sodium morrhuate as inducing agent)	8 weeks	To develop chronic mandibular osteomyelitis in miniature swine	Clinical evidence of mandibular osteomyelitis was developed in all mini swine by eight weeks post-infection. At this time, *S. aureus* was recovered from all six mini swine where bone wax had been used to seal the trephine hole, but not from the two PMMA animals	[131]
Large White	F	45.4 kg	*S. aureus* ATCC 29213. 10^7^ CFU	Experimental osteomyelitis in a model of gunshot fracture	Tibia	14 days	To create a model of ballistic wounding in the proximal tibia of pigs	The incidence of osteomyelitis was significantly reduced thanks to treatment with antibiotics. The histological examination confirmed the diagnosis of osteomyelitis based on the presence of purulent material inside associated bone and osteonecrosis.	[220]
Large White × Pietrain male castrated	na	50–65 kg	*S. aureus* (field strain isolated from a human orthopaedic infection). 1.2 × 10^3^ CFU	Orthopaedic implant–associated infection	Tibia	28 days	To create cDNA libraries that reflected changes in immune cell function after exposure to infection with *Staph. aureus*	7620 ESTs were clustered into 1029 clusters with an average of 3.6 sequences and 3846 singletons.	[223]
Yorkshire–Landrace crossbred	F	8 weeks, 20–25 kg	*S. aureus* strain S54F9 (clinical strain isolated from a chronic embolic porcine lung abscess). 2 × 10^9^–2.5 × 10^9^ CFU once (group 1 and 2) or twice (group 3 and 4) at 0 h and 12 h.	Non–traumatic osteomyelitis	Ear vein	6–12–24–48 h	To evaluate the pig as a model for the development of osteomyelitis following haematogenous spread of *S. aureus*	Disseminated micro abscesses within the lungs by 6 h were developed (but disappeared at 48 h). Within bones, lesions were localized in separate foci.	[142]
Yorkshire–Landrace crossbred	F	8–9 weeks, 15 kg;	*S. aureus* strain S54F9 (field strain isolated from a chronic embolic porcine lung abscess). 75, 7.5 × 10^2^, 7.5 × 10^3^, 7.5 × 10^4^, 7.5 × 10^5^ CFU	Acute haematogenous localized osteomyelitis	Brachial artery	5–15 days	To develop a porcine model for haematogenous localized osteomyelitis	Any lesion was not developed in low dose infection models. Pigs inoculated with 5000 and 50,000 CFU ⁄ kg BW only developed micro abscesses in bones of the infected legs. In trabecular osteonecrosis, bone lesions were evident.	[224]
Yorkshiree Landrace–cross pigs	F	12-weeks, 30 kg	*S. aureus* strains UAMS–1 (isolated from a case of human osteomyelitis), NCTC–8325–4 (isolated from a case of human sepsis) and S54F9 (isolated from a chronic embolic pulmonary abscess in a Danish slaughter pig). 3 × 10^5^ CFU	Haematogenous osteomyelitis	Ear vein	11 or 15 days	To compare the infection potential of the porcine strain (S54F9) with two *S. aureus* strains of human origin (UAMS–1 and NCTC–8325–4) in this model and to examine the development of HO with a focus on pathology and the localization and microenvironment of *S. aureus*	In three, one, and none of the recipients of porcine and human strains, respectively, bone lesions were present. On the CT scans, the vascularized bone tissue was seen as foci of increased opacity.	[225]
Yorkshire–Landrace crossbred pigs	F	12 weeks, 30 kg	*S. aureus* S54F9. 3 × 10^5^ CFU	Haematogenous osteomyelitis	Femoral artery	11–15 days	To describe a new intra–arterial inoculation technique in a porcine model of juvenile haematogenous osteomyelitis	Percutaneous catheterization is not an option due to the depth of the artery’s position. This model provides a reliable method for detecting lesions that discriminates the naturally occurring HO in long bones.	[150]
Specific pathogen–free	na	12 weeks, 30 kg	*S. aureus* S54F9. 1.5 × 10^7^ CFU (pig no.1) 1.5 × 10^8^ CFU (pig no.2)	Haematogenous osteomyelitis	Femoral condyle	6–8 days	To examine the histological bone changes of experimentally induced osteomyelitis in the porcine model	Lesions found in animals resemble those found in children suffering from haematogenous osteomyelitis	[226]
Danish Landrace	F	67–77 kg	*S. aureus* strain S54F9, (spa type t1333). 10^4^ CFU	Implant–associated osteomyelitis	Tibia	5 days	To investigate cefuroxime penetration during implant–associated osteomyelitis	In the implant cavities, lesions referable to bone destruction were found; no alteration in adjacent areas was noted. Cefuroxime penetration into infected bone was incomplete.	[227]
Danish SPF Landrace	F	3–8 months	*S. aureus* strain S54F9 (spa–type t1333). Low dose (10^2^ and 10^3^ CFU) High dose (10^4^ CFU)	Implant associated osteomyelitis	Tibia	5 days	To describe a novel porcine implant associated osteomyelitis model.	A significantly higher volume of bone lesion, number of neutrophils, concentration of acute–phase proteins in serum and enlargement of regional lymph nodes were induced by a high inoculum. Therefore, a threshold of 40 neutrophils for 10 high power fields was considered for the histopathological diagnosis of high–grade IAO.	[219]
Landrace SPF	F	35 kg	*S. aureus* S45F9. 10^4^ CFU	Implant–associated osteomyelitis	Tibia	2–4–6 days	To elucidate how deep implant–associated osteomyelitis can go into the peri–implanted bone tissue within a week	On implants and from 25 µm to 6 mm into pathological bone area *S. aureus* bacteria were identified.	[228]

Abbreviations: na = not available.

## Data Availability

Not applicable.
